# Viologen-Based Electrochromic Materials: From Small Molecules, Polymers and Composites to Their Applications

**DOI:** 10.3390/polym11111839

**Published:** 2019-11-08

**Authors:** Kwok Wei Shah, Su-Xi Wang, Debbie Xiang Yun Soo, Jianwei Xu

**Affiliations:** 1 Department of Building, School of Design and Environment, National University of Singapore, 4 Architecture Drive, Singapore 117566, Singapore; 2 Institute of Materials Research and Engineering, A*STAR (Agency for Science, Technology and Research), 2 Fusionopolis Way, Innovis, #08-03, Singapore 138634, Singapore; wangs@imre.a-star.edu.sg (S.-X.W.); debbie-soo@imre.a-star.edu.sg (D.X.Y.S.); 3 Department of Chemistry, National University of Singapore, 3 Science Drive 3, Singapore 117543, Singapore

**Keywords:** viologen, electrochromic, electrochromic device, viologen-based polymer

## Abstract

Organic materials have gained considerable attention for electrochromic (EC) applications owing to improved EC performance and good processability. As a class of well-recognized organic EC materials, viologens have received persistent attention due to the structural versatility and property tunability, and are major active EC components for most of the marketed EC devices. Over the past two decades, extensive efforts have been made to design and synthesize different types of viologen-based materials with enhanced EC properties. This review summarizes chemical structures, preparation and EC properties of various latest viologen-based electrochromes, including small viologen derivatives, main-chain viologen-based polymers, conjugated polymers with viologen side-chains and viologen-based organic/inorganic composites. The performance enhancement mechanisms are concisely discussed. The current marketed viologens-based electrochromic devices (ECDs) are briefly introduced and an outlook on the challenges and future exploration directions for viologen-based materials and their ECDs are also proposed.

## 1. Introduction

**Figure 1:**
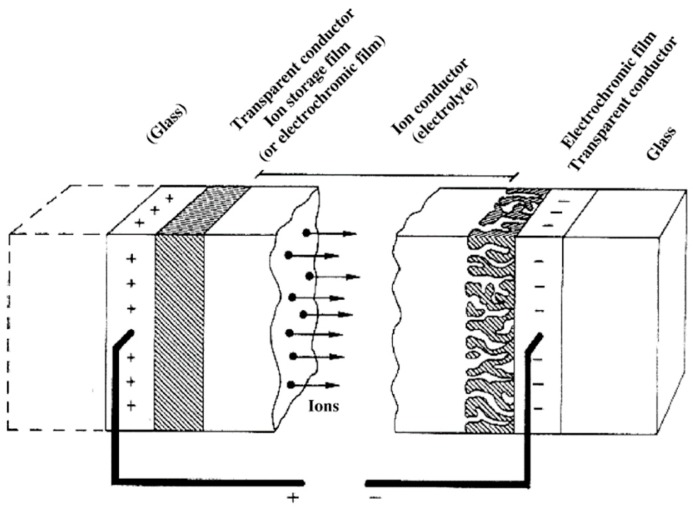
Basic design of an electrochromic device, showing transport of positive ions under the action of an electric field. Reprinted with permission from Elsevier Ltd. [13].

Over the past two decades, electrochromic (EC) materials have attracted significant research interest and have been extensively investigated for potential applications in smart windows [1,2,3,4,5,6], rearview mirrors [7], glare reduction [8], low power consumption displays [9,10]. An EC material has the ability to change its optical color (transmittance and/or reflectance) reversibly upon applying a suitable bias which causes electrochemical oxidation or reduction [11,12]. The change in color usually takes place between a transparent (bleached) state and a colored state, or between two or multiple colored states. Figure 1 presents the basic structure of an electrochromic device (ECD) which comprises two EC material films bounded to conductive electrodes, a conductive electrolyte for electron and ion transfer, as well as glass substrates on both sides of the device [13]. The performance of ECDs is highly dependent on the electrochrome employed, the choice of electrolyte and the entire device assembly. There are two types of ECDs depending on the modes of device operation, namely, the transmission mode and the reflectance mode. The transmittance mode employs transparent conductive electrodes, which can control the light intensity passing through. A typical example of transparent EDCs is smart windows which can automatically adjust solar light penetration to improve indoor comfort. By contrast, in the reflectance mode, one of the transparent conducting electrodes is replaced with a reflective surface, which controls the intensity of reflective light. The widely used self-darkening electrochromic car mirror which can regulate reflections of flashing light from following vehicles is a typical example of reflective ECDs.

Generally, EC materials can be mainly grouped into inorganic and organic categories. Inorganic EC materials include transition metal oxides (e.g., V_2_O_5_, WO_3_, MoO_3_, Nb_2_O_5_, Ir(OH)_3_ and NiO_x_H_y_) and inorganic coordination complexes (e.g., Prussian blue), [14,15]. Organic EC materials are π-conjugated organic molecules or polymers with one of the redox states in a free-radical form, such as 1,1′-disubstituted-4,4′-bipyridinium salts (viologens), conducting polymers (e.g., polyaniline, polythiophene), anthraquinone, aromatic imides and triphenylamine [16,17,18]. In comparison with their inorganic counterparts, organic EC materials exhibit unique advantages including good processability, abundant colors with readily tunable EC properties through structural modifications.

**Figure 2:**
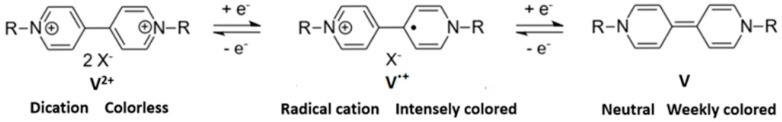
Different redox states of viologen.

Viologens, 1,1′-disubstituted-4,4′-bipyridinium salts, are by far the most intensively investigated small molecule-based EC materials for both research and commercial application purposes. The major advantages of these materials reside in their good optical contrast, high coloration efficiency, redox stability, ease in molecular design and the feasibility for large-area preparation. The EC behaviors of viologens were first reported by Michaelis and Hillin in 1933, since they found 1,1′-dimethyl-4,4′-bipyridinium (methyl viologen) was violet in a reduced state [19]. As shown in Figure 2, viologens possess three reversible redox states: a dication (V^2+^), a radical cation (V^•+^) and a neutral form (V). Viologens can be synthesized as small molecules or be incorporated into polymer backbones, or as functional attachments to a polymer chain. The EC properties of viologens can be readily tuned through altering nitrogen substituents and various counter-anions. In addition, the colorless dication species of viologens can be prepared as air-stable salts, and their intensely colored radical cations are considered among the most stable organic radicals as well. 

Despite the long history of electrochromism since the 19th century, it was not until the end of last century that we witnessed its considerable scientific and technological progress. Up to now, the most successful commercial applications of EC materials are the widely used anti-glare rare-view car mirrors invented by Gentex Corporation and the auto-dimming smart windows installed on Boeing 787 Dreamliner, both of which employ viologens as the major active EC components. Over the last two decades, extensive efforts have been made to improve the EC properties of viologens, including modification of small molecular viologens and polymeric viologens, incorporation of viologens into conjugate polymers and construction of polymer composite materials with inorganic nanofillers etc. This review summarizes recent development in viologen-based electrochromes, including structure, preparation, EC performance of viologen-based ECDs and property enhancement mechanisms. Finally, commercialized viologen-based ECDs are briefly described and an outlook on the current challenges of viologen-based EC molecules and polymers as well as future exploration is also suggested. 

## 2. Viologen-Based Electrochromes 

Electrochromes can be classified into three basic types according to the solubility of their redox states. Type I materials are soluble in the electrolyte solution under both reduced and oxidized states, an example of which is methyl viologen. Type II materials, such as 1,1′-di-heptyl-4,4′-bipyridilium (heptyl viologen), are soluble in the colorless state, but can form a solid film on the surface of the electrode under the coloration state. Type III materials, such as polyviologens and viologen-functionalized conjugated polymers, are in solid forms under all the redox states, and such materials are usually employed as thin films on the electrode surface. In cases of types II and III materials, once the redox state has been switched, the material can retain the color without additional injection of charge, which is called “memory effect”. However, for type I materials, it is necessary to keep the current flowing until the whole solution is electrolyzed because the newly formed soluble state may diffuse away from the electrode. In this section, different types of viologen-based EC materials are introduced and the performance of their corresponding ECDs is discussed. 

### 2.1. Small Molecular Viologens 

**Figure 3:**
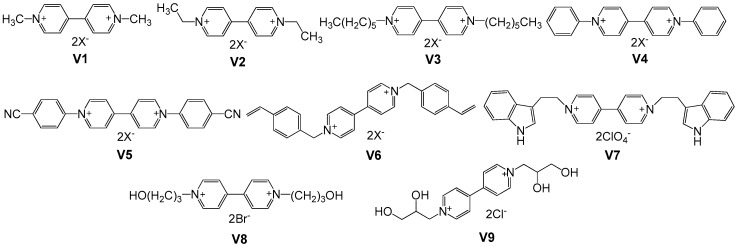
Structures of small molecular viologens with various substituents at nitrogen positions.

The molecular design and synthesis of viologens with different subsituents are facile and straightforward owing to the basicity of the nitrogen atoms of 4,4′-bipyridine, which can undergo an SN2 reaction with organic halides, giving rise to di-substituted cationic species with the halide as the counteranion. The common halide anions can be further converted to PF_6_^−^, ClO_4_^−^, BF_4_^−^ or OTf^−^ via an anion exchange process. Figure 3 shows the structures of common small molecular viologens with various subsituents at nitrogen positions. The ECD performance of selected small viologen molecules and their derivatives discussed in this paper is summarized in Table 1.

**Figure 4:**
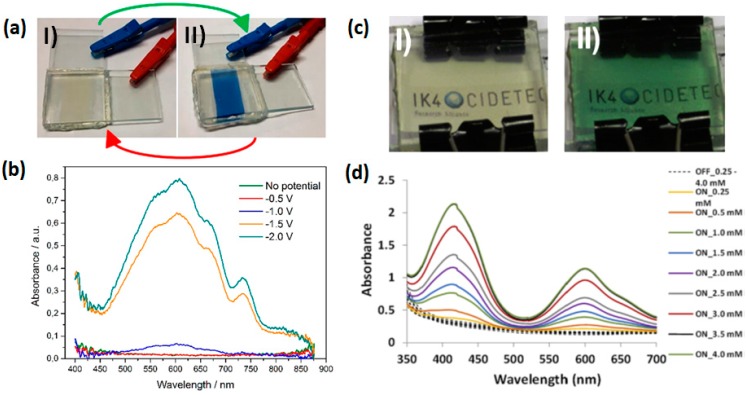
(**a**) The contrast color of ethyl viologen-based ECD at bleached state (I) and colored state (II). (**b**) The UV−vis spectra of ethyl viologen-based ECD at different potentials. (**c**) The contrast color of cyanophenyl viologen based ECD at bleached state (I) and colored state (II). (**d**) The UV−vis spectra of ECDs containing different concentrations of cyanophenyl viologen at their radical states. Reproduced with permission from American Chemical Society [29] and Elsevier Ltd. [20].

Generally, an intense color is generated upon reduction of the viologen dication, leading to a strong absorption at its radical cation state. The color of this reduction state mainly depends on the substitution groups on the nitrogen of the bipyridinium salt. As illustrated in Figure 4a,b, the absorption spectra of the radical cation of ethyl viologen (**V2**) exhibit a strong absorption peak close to 608 nm and weak absorption at near-UV irradiation (400–450 nm) region, resulting in a blue color upon reduction [29]. Other viologens substituted with alkyl chains, such as methyl viologen (**V1**) [30], heptyl viologen (**V3**) [31], benzyl viologen [32] and vinyl benzyl viologen (**V6**) [33], all show similar optical characteristics to ethyl viologen with the absorption peak around 600 nm at the radical cation state, leading to a typical blue or violent-blue color. However, when the nitrogen in the bipyridinium ring is substituted by aryl groups, such as phenyl viologen (**V4**) [34] and cyanophenyl viologen (**V5**) [20], the resulting viologens would exhibit a green color upon reduction. As presented in Figure 4c,d, cyanophenyl viologen shows two strong bands with a maximum of absorption at 420 and 600 nm. It should be noted that viologens can undergo two reversible one-electron reduction steps to form either radical cations or neutral species with different colors. For example, the radical cation of cyanophenyl viologen can be further reduced to neutral state which exhibits a red color with a single wide absorption band around 500 nm [20]. It has been found that the employed electrolytes also have influence on the coloration and write–erase ability of the viologens [35]. In aqueous solution, the neutral state of alkyl viologen (V^0^) would react with V^2+^, resulting in the generation of undesirable dimerized viologen radical cation (2V^+●^), leading to a crimson color and poor write–erase ability because of the irreversible comproportionation [31]. In contrast, the dimerized viologen radical cation would spontaneously dissociate in most organic solvents owing to their relatively weak solvation energy [36], and it has been demonstrated the formation of dimerized viologen could be controlled in ionic liquid-based electrolytes [37]. Moreover, bulky substituents of viologens which create hindrance among molecules can reduce the possibility of dimerization or aggregation, thus enhancing the cyclic stability of the corresponding ECDs [21]. 

Recently, Sydam et al. prepared a novel viologen electrochrome 4,4′-bipyridinium diperchlorate (**V7**) comprising a 4,4′-bipyridyl core sandwiched between two indole moieties [22].Usually, the radical cations of bipyridyl system upon reduction are likely to form radical dimers due to the aging and slow re-oxidation process. Here, due to the bulky indole groups attached to the 4,4′-bipyridyl system, the dimerization of radical cations is not favored due to the steric hindrance. Moreover, the indole groups can also donate π-electron density to the bipyridyl system, leading to a faster color bleach process (re-oxidation of radical cation species) of this electron-rich viologen. The ECD of **V7** exhibited rapid coloration and bleaching times of ~2 s, at a frequency of 0.33 Hz. The overall write–erase efficiency was determined to be 95, 86, and 79% at switch durations of 3, 5, and 10 s, respectively, demonstrating the high efficiency of the system during fast switching.

It is well known that hydroxy is a typical auxochrome. Pan et al. recently prepared a viologen molecule with hydroxy groups as substituents (**V8**, Figure 3), which was employed to fabricate a single-layer all-in-one ECD with polymer gel electrolyte [23]. The resulting ECD of **V8** exhibited a low driving voltage (0.9 V), a remarkably high optical contrast up to 82% and an excellent coloration efficiency of >240 cm^2^ C^−1^. Later, the same group developed bis(dihydroxyalkyl) viologen (**V9**), and its ECD demonstrated even a lower coloration voltage down to −0.6 V and exhibited a high coloration efficiency up to 301 cm^2^ C^−1^ [24]. The optical contrast of it only reduced by 1% after consecutive switch on and off for 10,000 cycles between −1.0 V and 0 V, demonstrating an excellent long-term cycling stability of this device.

**Figure 5:**
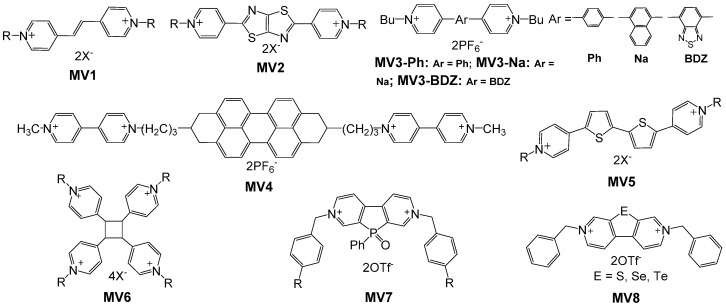
Structures of various viologen derivatives with a bridging group or a spacer between two pyridyl groups.

Apart from the variations on the substituents at nitrogen positions, great efforts have also been devoted to modifying viologens to further fine-tune the EC properties and impart additional functionality. The introduction of bridging groups and heteroatoms into the viologen molecules can enhance the electronic and photophysical properties of viologens. The chemical structures of a variety of modified viologen derivatives with a bridging group or a spacer between two pyridyl groups are shown in Figure 5.

Deng et al. developed a variety of vinylbipyridinium derivatives (**MV1**) to create a greater π-conjugated system of viologen with red-shift of the absorption bands at reduced states [25]. The corresponding ECDs exhibited highly reversible colorless-to-magenta/pink electrochromism with cyclic stability over 10,000 times, and the coloration and bleaching time could reach ~1 s. The fast response time could be ascribed to the improved charge or ion transportion of the conjugated system during the redox process. In addition to improving the switching time, the introduction of conjugated moieties between the two pyridine rings can also induce fluorescence to the system. Shi et al. prepared a new series of aryl-bridged viologens (**MV3**) by introducing benzene, naphthalene, anthracene and benzothiadiazole rings by Suzuki–Miyaura coupling [26]. These viologen derivatives were found to possess electrochromic-fluorescent dual functionality due to an increased conjugated degree of π-framework. ECDs consisted of **MV3-Ph**, **MV3-Na** and **MV3-BDZ** with ferrocene as the counter electrode exhibited a high transmittance change of 76.9%, 74.1% and 69.0% at various wavelengths and fast response times within 9.5, 18.4 and 24.0 s, respectively. Good stability over 1000 cycles was observed with all the three electrochromes. Attributing to the large conjugated system, the viologens bearing benzene, naphthalene, anthracene and benzothiadiazole rings emitted intense blue, brilliant blue, yellow and blue fluorescence, respectively, in bleached states. Thiazolothiazole-bridged viologens (**MV2**) [38] and thienoviologens (**MV5**) [39] were also prepared and displayed strong fluorescence and reversible electrochromism.

Multicolor electrochromism of viologen-based materials can be realized through introduction of electroactive moieties. Kim and co-workers developed a new electrochromic perylenediimide (PDI)-viologen dyad (**MV4**) by integrating PDI and two viologen units [27]. The tetracationic **MV4** with high molecular weight can easily adhere to negatively charged poly(3,4-ethylenedioxythiophene)–poly(styrenesulfonate) (PEDOT:PSS) to form multilayer films via layer-by-layer (LBL) assembly. Since both PDI and viologen exhibit intense absorption bands in the visible region after electrochemical reduction, the prepared LBL multilayer films showed multiple-color electrochromism ranging from red to blue. They also fabricated a dual-type ECD employing the LBL films with poly(3-hexylthiophene) (P3HT) film as a counter electrode. The resulting device showed intense color switching between deep red and dark blue by alternating the applied potential.

An effective way to improve the switching time of EC material is to enhance the electron transport rate within the system. Very recently, Ma et al. synthesized a series of cyclobutane viologen derivatives (**MV6**) from trans-1,2-bis(4-pyridyl)ethylene under UV radiation [28]. When using ferrocene as the counter redox material, the ECDs based on **MV6** exhixit rapid response times up to 0.48 and 0.79 s for the coloring and bleaching processes, respectively. Compared to other pyridinium salt EC materials which usually require a response time longer than 2 s, the fast response of ECD based on these cyclobutane derivatives could be attributed to the highly symmetric structure from the expanded shared electrons within the four pyridinium rings, making electron transfer process much easier during redox processes. 

In recent years, several groups have made efforts to combine heteroatoms with viologens to tune their electrochemical and optical properties. In 2015, Stolar et al. reported a series of *N*-benzylated phosphoryl-bridged viologen analogues (**MV7**) [40]. **MV7** exhibit similar EC properties to methyl viologen. However, the reduction threshold for both redox steps was 500 mV lower than that for methyl viologen due to the addition of the central five-membered phosphole oxide ring. The increased tendency for electrochemical reduction of **MV7** could be explained by the significantly lowered LUMO level arising from effective σ*−π* hyperconjugation [41]. Furthermore, they introduced a series of substituted phenyl substituents with either electron-donating or electron-withdrawing groups at the para position for tuning the electronic properties of the phosphaviologens. Even at the lower reduction potentials, the various redox states still could preserve their colors (colorless, blue/purple, orange) in each of the species. Very recently, Li et al. developed a series of electron-accepting chalcogen-bridged viologens (**MV8**) through incorporation of chalcogenophenes to viologen [42]. It was found that the heavy atom substitution (S, Se and Te) could tune the optoelectronic properties of these scaffolds through narrow the HOMO–LUMO bandgaps along with increased HOMO levels, which caused the redshift of the absorption bands to the visible region. Furthermore, the introduction of chalcogen could also facilitate the reduction process via decreasing the energy of the LUMO-levels, leading to significantly lowered reduction potentials compared to those of the starting materials. These new materials demonstrated great potential for further applications in low-voltage and energy-effiecient ECDs. 

### 2.2. Polyviologens

**Figure 6:**
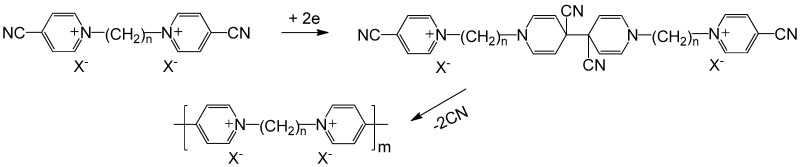
Electropolymerization of bis(4-cyano-1-pyridino)alkanes to polyviologens.

Early application of viologen was focused on aqueous electrolyte display systems. However, the leakage happened in solution type devices was a potential stability problem. One of the solutions is to immobilize viologen onto electrode surfaces through the formation of thin films by electropolymerization. Higher switching speeds and longer life cycles are the main advantages of these thin film-type polyviologens. The reductive electropolymerization of bis(4-cyano-1-pyridino) alkenes is a well-known and simple approach to synthesize polyviologens. The mechanism of this reductive electropolymerization is proposed in Figure 6, including the formation of a dimer and followed by loss of two cyanide ions [43]. Upon reduction, polyviologens bearing various substituents like butyl [44], heptyl [44], decyl [44], xylenyl [45] can be obtained. It has been demonstrated that the alkyl chain length as well as the choice of the counter-ion type in the electrolyte during polymerization can notably affect the morphology, spectral and electrochemical properties of the polyviologen films [43,46,47]. In an early work in 1993, Saika et al. prepared bis(4-cyano-1-pyridinio) derivatives which were subsequently deposited on electrodes by cathodic electropolymerization. The deposited polyviologen films showed characteristic electrochromisms with colorless or slightly yellow to blue- or red-violet [44]. In 2003, DeLongchamp et al. reported a ultra-high-contrast (with a transmittance change (ΔT) up to 82.1% at 525 nm) EC composite material through LBL assembly of cathodically colored poly(hexyl viologen) and PEDOT : PSS colloid [48]. In 2008, Ho et al. presented an all-solid-state ECD based on cathodically colored poly (butyl viologen) and anodically colored Prussian blue confined to the electrode surfaces [49]. This all-solid-state ECD showed a high optical contrast (ΔT up to 65%) with a coloration efficiency of ca. 163 cm^2^/C at 650 nm and good stability during 4000 cycles. 

**Figure 7:**
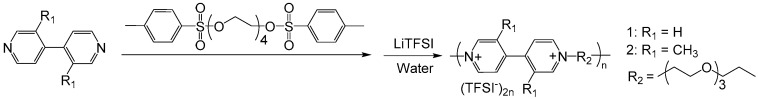
Synthesis of polyviologens via Menshutkin reaction.

Apart fromelectropolymerization, a variety of polyviologens containing bromide or tosylate counter-ions have been synthesized via Menshutkin reactions of 4,4′-bipyridine and the corresponding alkyl halides or alkyl ditosylates [50,51,52,53,54,55]. In 2017, Sato et al. reported the synthesis of dimethyl-substituted polyviologen with polyethylene glycol backbones by Menschutkin reaction (Figure 7) [56]. It was found that the introduction of methyl groups into the bipyridine scaffolds could alter the stereostructure and shift the color of the corresponding cation radicals from purple to blue. Moreover, the decloration response rate was improved by a factor of 13 by relaxing excessive π-stacking between the viologen moieties, as evidenced by density functional theory calculation.

Very recently, Gao et al. synthesized a UV–curable acrylate-functional viologen to deposite polyviologen films on indium tin oxide (ITO) electrode surface [57]. The simple and scalable UV curing approach resulted in polyviologen films with good chemical stability and excellent adhesion between the polymer film and the ITO glass. It was found the corresponding ECD with gel electrolyte exhibited good contrast (ΔT~39.5%), moderate response time (11–13 s) and excellent color change reversibility from deep blue to pale yellow under an applied potential of −2.0 V and 2.0 V, respectively.

**Figure 8:**
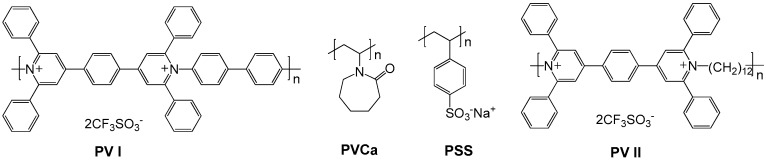
Chemical structures of poly(pyridinium) salts and their polymer additives.

In addition to the aforementioned poly(alkyl viologens), the polyviologens with rigid-rod structures (aromatic poly(pyridinium salt)s) are synthesized by polymerization of 4,4′-(1,4-phenylene)bis(2,6-diphenylpyrylium salts) with aromatic diamines [58,59]. This class of poly(pyridinium) salts have been demonstrated to possess good mechanical properties and film forming abilities, and they exhibit EC behavior with color changing from transparent slight yellow to blue [60]. Petrov and coworkers recently focused their efforts on the EC property study of the aromatic poly(pyridinium triflate) and its complexes which were obtained by noncovalent modification with nonconductive additive poly(*N*-vinylcaprolactam) (**PVCa,** Figure 8) [61] and poly(styrene sulfonate) (**PSS**, Figure 8) [62]. It was found the polymer interactions in the formed complexes such as hydrogen bonds, electrostatic interactions and van der Waals forces could lead to changes in EC contrast and switching time of the poly(pyridinium triflate). The same research group also systematically investigated the EC properties of poly(pyridinium triflates) films in various aqueous electrolyte (KCl, KBr, LiCl, NaCl) solutions [63]. It was found that as the hydrated cation size increases in the row of of Li^+^, Na^+^ and K^+^, replacing K^+^ by the more mobile Na^+^ and Li^+^ could improve bleaching time and deteriorate both coloration time and optical contrast due to the obstructed cation transport in the redox reactions. By contrast, replacing Cl^−^ by Br^−^ anions did not affect the EC characteristics, suggesting the anion transport is suppressed due to inability of bulky organic counterion triflates (CF_3_SO_3_^−^, which were initially doped in the polyvilogen films) to take part in the ion exchange between the film and electrolyte. They also studied the effect of polymer chain conjugation on the EC properties of the poly(pyridinium) salts. The EC behaviors of two poly(pyridinium triflate)s containing flexible non-conjugated alkyl spacer (**PV II**, Figure 8) and rigid-rod π-conjugated phenylene linkages (**PV I**, Figure 8) were comparatively investigated by a three-electrode cell and smart window prototypes [64]. Both polymers demonstrated reversible redox process. However, the introduction of the alkyl spacer leads to deterioration in the optical contrast and switching time due to the charge transport deterioration in the non-conjugated polymer backbone.

### 2.3. Viologen-Functionalized Conjugated Polymers

In the past two decades, conjugated polymers have gained significant research interest in the EC field due to their low cost, easy preparation, good processability, long term stability and color versatility [17,18,65,66,67]. Recently, considerable effort has been devoted to modifying the electronic properties of conjugated polymers through incorporation of additonal electroactive groups to the polymer matrix. It has been demonstrated that functionalization using redox active viologens with sharp color changes could effectively enhance the optical contrast of the polymer. Furthermore, when a conjugated polymer and viologen show different EC colors and their redox-potential ranges do not overlap one another, the resulting viologen-functionalized polymer is expected to present combined multi-electrochromism, which is more plentiful than that from their individual materials [68]. The preparation strategy of such viologen-functionalized conjugated polymers can be divided into two categories: incorporation of viologen units as part of the main chain or attachment to the side chain as a pendant, which will be elaborated in the following sections. ECD performance of selected polyviologens is summarized in Table 2.

#### 2.3.1. Viologen in the Main Chain

**Figure 9:**
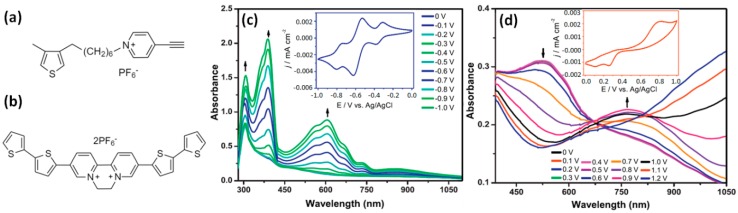
(**a**) The chemical structure of Th-CN monomer. (**b**) The chemical structure of the bt2dq^2+^ monomer. In situ UV–Vis specta for the PTh-V film deposited on ITO electrode inBmimPF6 recorded under different applied potentials from (**c**) 0 V to −1.0 V and (**d**) 0 V to 1.2 V in steps of 0.1 V. Readapted with permission from Elsevier Ltd. [69,79].

Polythiophene (**PTh**) is one of the extensively investigated conjugated polymers with strong electrochromism and diverse electrochemical properties [73,74,75,76,77]. Through chemical functionalization of the thiophene monomer with a cyanopyridine group, the thiophene backbone can be crosslinked with viologens to impart electrical conductivity as well as redox properties. Gadgil and co-workers reported the electrochemical polymerization of a cyanopyridine-functionalized thiophene (**Th-CN**) monomer (Figure 9a) to generate a conjugated polymer containing viologen cross-linked polythiophene (**PTh-V**) [69,78]. In situ spectroelectrochemical studies showed that the EC contrast of the polymer film was enhanced compared to that of the polythiophene film, and multi-electrochromism was achieved due to the incorporation of viologen groups As shown in Figure 9c,d, the copolymer exhibited coloration characteristics of both viologen and PTh moieties. The yellowish blue polymer turned dark blue (with a color efficiency of ca. 97 cm^2^ C^−1^ at 625 nm) during the reductive sweeping (0 to −1.0 V) and to transparent violet (with a color efficiency of ca. 12 cm^2^ C^−1^ at 770 nm) upon the oxidative sweeping (0 to 1.2 V). Krompiec et al. also prepared a diquat-quaterthiophene alternating EC copolymer with a viologen-like moiety in the main chain through electropolymerization of a novel bis(bithiophenyl) derivative of diquat (bt2dq^2+^, Figure 9b) [79].

#### 2.3.2. Viologen as Pendant

**Figure 10:**
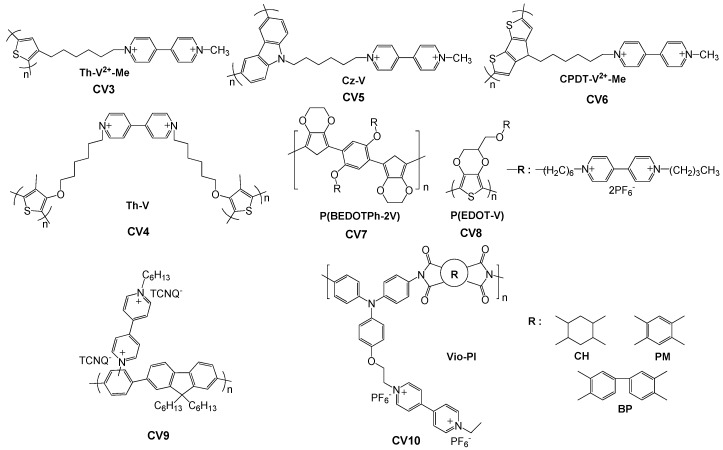
Conjugated polymers bearing viologen groups as pendant.

Compared with the aforementioned crosslinking method for integration of viologens into the polymer main chain, the attachment of viologen groups as pendants to the polymer backbone is a much more popular strategy for functionalization of conjugated polymers. As shown in Figure 10, viologen moieties have been anchored to a wide variety of conjugated polymers through chemically attachment to the monomers followed by electropolymerization, including polythiophene derivatives (**CV3**, **CV4**) [70,80], polycarbazole (**CV5**) [81], poly(cyclopentadithiophene) (P(CPDT),**CV6**) [82], poly [83] (P(BEDOTPh), **CV7**) [83], poly (3,4-ethylenedioxy-thiophene) (PEDOT, **CV8**) [71], polyphenylenes (PP, **CV9**) [84], polyimide (PI, **CV10**) [72] and so on. 

**Figure 11:**
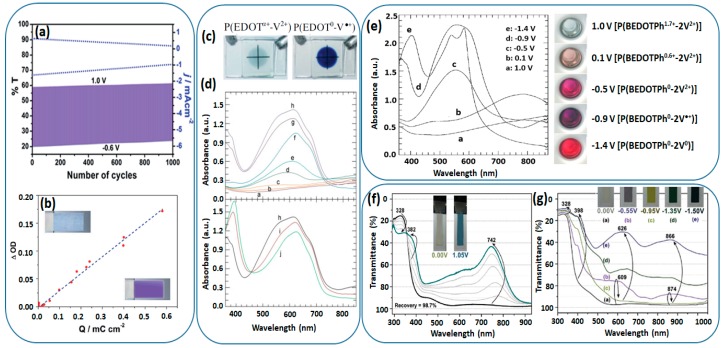
(**a**) Cycling stability test for electrochromic PTh-V at bleached (1.0 V) and colored (−0.6 V) states. The dotted line refers to the current change, and the solid line below refers to the transmittance change. (**b**) Optical density change (DOD) of the PTh-V film at 610 nm as function of charge density (Q) at different applied potentials ranging from 1.0 to −0.6 V. (**c**) Photograph of an ECD based on P(EDOT-V) film. (**d**) Spectroelectrochemical graphs of P(EDOT-V) film on ITO glass at various applied potentials: (a) 0.5 to 1.2 V, (b) 0.2 V, (c) 0 V, (d) −0.2 V, (e) −0.4 V, (f) −0.6 V, (g) −0.7 V, (h) −0.8 to −1.0 V, (i) −1.2 V and (j) −1.4 to −1.6 V versus Ag/Ag^+^. (**e**) Electronic absorption spectra of the P(BEDOTPh-2V) film on ITO glass at various potentials. Spectroelectrochemical graphs of Vio-BP film on ITO glass at different applied potentials from (**f**) 0.00 to 1.05 V and (**g**) 0.00 to −1.50 V. Readapted with permission from The Royal Society of Chemistry [70] and WILEY-VCH [71,72,83].

Polythiophene is a representative class of conjugated polymers as active components in optoelectronic devices due to their good processability as well as excellent thermal and electrochemical stability. Substitutions at the 3- and/or 4-position in the thiophene ring can minimize the occurrence of couplings during chemical or electrochemical polymerization, producing more regular structures and resulting in better properties. In an effort to develop EC materials with multi-electrochromism, Ko et al. introduced a methyl viologen to the 3-position of polythiophene via a long alkyl chain (**CV3**, Figure 10) [80]. Gadgil and co-workers functionalized an electropolymerizable 3,4-disubstituted thiophene monomer with a viologen unit connected to the side chain at the 3-position of the thiophene ring (**CV4**, Figure 10) [70]. The obtained viologen bearing polythiophene (**PTh-V**) exhibited excellent reversibility from almost transparent to dark violet with a good optical contrast (ΔT~39%) and a high coloration efficiency of ca. 305 cm^2^ C^−1^ at 610 nm (Figure 11a,b). Moreover, compared to the small molecular viologen, the conjugated PTh-V redox polymer also showed improved thermal stability due to the incorporation of polythiophene backbones. 

PEDOT is an electrochemically active polymer which shows a color change from dark blue in the neutral state to transparent sky blue when oxidized; however, the EC contrast is not satisfactory for practical applications. Since both PEDOT and viologen are cathodically colored, Ko et al. introduced a viologen unit to the EDOT monomer [71]. After electropolymerization, the obtained conjugated polymer (PEDOT-V) could reach a maximum transmittance change of 65% at 610 nm due to a synergistic electrochromism, which cannot be accomplished in the case of simple alkyl-substituted PEDOT derivatives (Figure 11c,d). The same research group also successfully integrated the viologen group into the electrochemically stable conductive polymer P(BEDOTPh), leading to **CV7** polymer films showing multicolored EC behavior with five distinct colored states (Figure 11e) [83]. 

Very recently, Yen and co-workers prepared novel solution-processable polyimides containing triphenylamine and pendant viologen moieties (**Vio-PI**) with strong donor–acceptor charge-transfer possesses write-once read-many-times memory behavior with excellent operation stability [72]. The obtained multicolored EC polymer films reveal ambipolar electrochemical behavior with a high optical transmittance contrast of coloration changed from transmissive neutral state to the cyan/magenta/yellow redox states (Figure 11f,g), implying great potential for application in smart windows and displays. 

### 2.4. Viologen Based Composite Materials 

#### 2.4.1. Composites with Metal Oxides 

In spite of their low cost and good processability, viologen-based materials are still lack of good electrical conductivity, cyclic stability and fast response times for practical applications. To further enhance their EC performance, hybrid organic-inorganic EC systems have been developed through chemical anchoring or adsorption of viologen moleculars onto various nanostructued semiconductors (such as TiO_2_, ZnO, ITO, SnO_2_ etc) with a high specific surface area and good crystallinity [85,86,87,88,89,90,91,92,93,94,95,96,97,98,99,100,101,102,103]. The improved switching speed, cyclic stability and contrast ratio of such a hybrid system is ascribed to the fast interfacial electron and ion transfer between the nanocrystalline electrode and the anchored chromophore together with sufficient coloration resulted from the high volume density of the tethered viologens [88]. Table 3 summarizes the ECD performance of various selected viologens-based composite materials.

**Figure 12:**
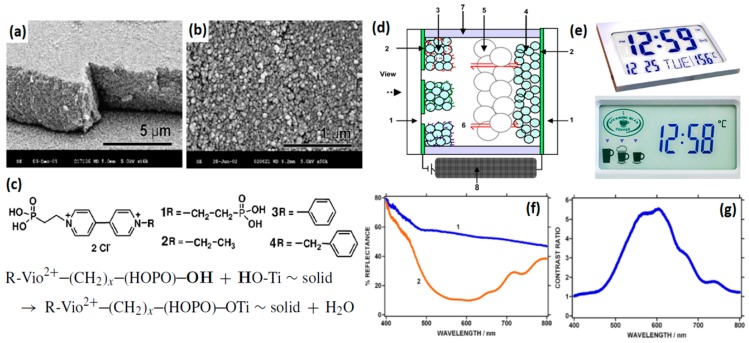
(**a**,**b**) Scanning electron micrograph (SEM) images of nanocrystalline TiO_2_ layers of 4 μm thickness. (**c**) Examples of viologen molecules functionalized with phosphonic acid groups for attachment onto TiO_2_ surface through formation of phosphonic acid ester. (**d**) Cross-section structure of a Nanochromics^TM^ display. 1: Transparent glass, 2: conducting glass coating, 3: viologen/TiO_2_, 4: SnO_2_:Sb counter electrode, 5: white reflector TiO_2_ layer, 6: electrolyte, 7: sealant, 8: electric driving system. (**e**) Examples of Nanochromics^TM^ displays. (**f**) Reflectance spectra of bleached 1 and colored 2 state vs. wavelength (electrode area 4 cm^2^ for both viologen/TiO_2_ (6 μm) and SnO_2_:Sb; applied voltages: 0 and −1.3 V, respectively.) (**g**) Contrast ratio vs. wavelength at −1.3 V. Readapted with permission from Elsevier Ltd. [96].

In particular, nanocrystalline TiO_2_ films (Figure 12a,b) are the most widely used semiconductor substrates for adsorption of viologens owing to their easy preparation, large surface area, good electrical conductivity, and good transparency to visible light and high surface affinity towards certain ligands [85,86,87,88,89,90,91,92,93,94,95,96,97]. For chemical attachment onto the TiO_2_ surface which bears OH groups, the employed viologen molecules are usually functionalized with at least one phosphonic acid group (-P(=O)(OH)_2_) linked to the viologen core atoms via a flexible alkyl chain ((CH_2_)_n_) (Figure 12c) [96]. As the chromophores are chemically anchored via the formation of phosphonic esters, the electrode reactions are less influenced by diffusion and thus a high-speed response becomes possible. In an early study, Cinnsealach et al. developed an EC window based on a viologen modified transparent nanostructured TiO_2_ film (anatase, 4.0 μm thick) electrode with conducting glass as the counter electrode. The derived device can reach a coloration efficiency up to 170 cm^2^ C^−1^ at 608 nm, a fast switching time of 1 s and an excellent stability over 10,000 test cycles, showing great promise for commercial application. In 2004, Choi et al. reported an ordered mesoporous nanocrystalline anatase TiO_2_ electrode with a high accessible surface area (up to 172 m^2^ g^−1^), which enables a considerably greater adsorption of viologen molecules (8.6 times higher than that of the conventional nanocrystalline TiO_2_ electrode), thus leading to an enhanced contrast ratio and redox activity. A “Nanochromics^TM^” EC display device was developed based on the viologen/nanocrystalline TiO_2_ composite electrode with SnO_2_: Sb as the counter electrode (Figure 12d–g). Such a device can achieve a high contrast ratio above 5 at around 600 nm and fast coloration–bleaching transition times from 10 ms to several seconds depending on the pixel size. Stable performance has been demonstrated for more than a million cycles without notable degradation.

**Figure 13:**
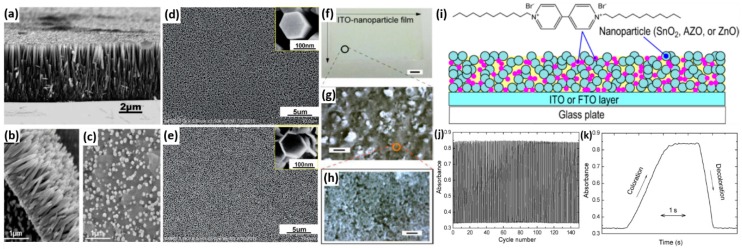
(**a**) SEM image of the ZnO nanowires array hydrothermally grown on ITO glass substrate. SEM image of (**b**) ZnO nanorods grown on ITO/polyethylene terephthalate substrate and (**c**) the ZnO nanorods modified with methyl viologen molecules. SEM images of (**d**) ZnO nanorods and (**e**) ZnO nanotubes hydrothermally grown on ITO glass substrates. (**f**) Photograph and (**g**,**h**) SEM images of a viologen/ITO nanoparticle electrode. Scale bars in the images are 1 mm, 10 μm, and 1 μm, respectively. (**i**) Schematic illustration of metal oxide nanoparticles/viologen composite film electrodes. (**j**) Coloration−decoloration cycles of the SnO_2_-DDV/FTO electrode in aqueous electrolyte between −0.55 and 0 V at a switching interval of 3 s monitored at a wavelength of 510 nm and (**k**) the extracted short-term cycles with absorbance plotted as a function of time. Readapted with permission from American Chemical Society [98,103], Elsevier B.V. [99,102] and The Royal Society of Chemistry [100].

In addition to TiO_2_, viologens have also been incorporated into other high-surface-area nanostructured semiconductor electrodes via physical adsorption, such as ZnO nanowire/nanorod/nanotube arrays hydrothermally grown on the ITO substrates (Figure 13a–e) [98,99,100] and commercial ITO nanoparticles dip-coated on ITO glass (Figure 13f–h) [17,18]. A sufficient color contrast ratio, a fast response time and good durability have been achieved in all those examples. In a recent study by Hoshino and co-workers, the EC properties of composite film electrodes consisting of viologen and different kinds of metal oxide (SnO_2_, Al-doped ZnO (AZO), and ZnO) nanoparticles have been investigated (Figure 13i–k) [19]. It has been demonstrated that the hydrophilicity of the nanospaces created by the nanoparticles is significantly important as they provide specific sites for ion transportation and electrochemical reactions. The coloring uniformity, coloring contrast and coloring/decoloring response times of the hybrid electrodes are determined by the nature of the nanopores and the affinity of the nanoparticles for the alkyl viologen as well as the current collector (ITO/fluorine doped tin oxide (FTO)). The optimal EC performance has been delivered by the SnO_2_-1,1′-didodecyl-4,4′-bipyridinium (DDV) composite film coated on FTO current collector, which exhibits a constant high absorbance contrast and fast coloration and bleaching times of 1.5 and 0.53 s, respectively.

#### 2.4.2. Composites with Carbon Nanomaterials

**Figure 14:**
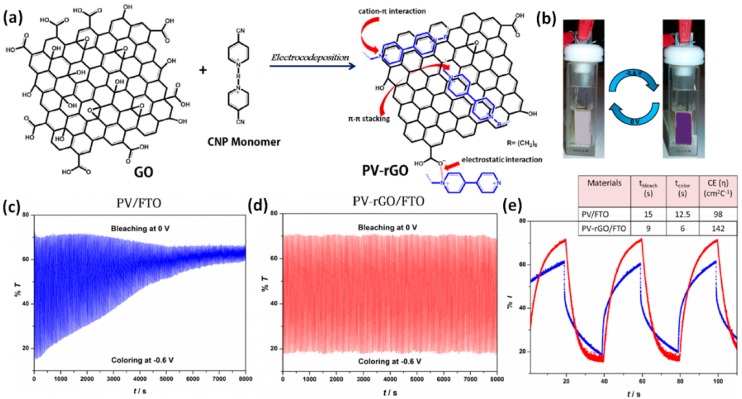
(**a**) Schematic of GO and its electrocodeposition with CNP monomer to yield PV–rGO nanocomposite and illustration of possible non-covalent interactions in PV–rGO structure. (**b**) Photographs of the electrochromic switching in its colorless and colored state. Voltage controlled transmittance changes at bleached (0 V) and colored (−0.6 V) states for PV/FTO (**c**) and PV–rGO/FTO (**d**) films at 525 nm in 0.1 M KCl aqueous solution. (**e**) Electrochromic transmittance response vs. time during potential switching of PV/FTO (blue line) and PV–rGO/FTO (red line) films between 0 (bleach) and −0.6 V (color) and the duration was 20 s. Inset table shows color switching times and coloration efficiency values for PV/FTO and PV–rGO/FTO composite films. Readapted with permission from Elsevier Ltd. [104].

Graphene is a promising and widely investigated two-dimensional material for electronic and optical applications owing to its unique properties such as large surface area, excellent transparency and flexibility, high electrical conductivity and carrier mobility, good chemical and thermal stability [110,111,112]. Despite the low cost and good processability, the cyclic reversibility and response times of viologen-based EC materials are still needed to be improved for practical applications. Incorporation of graphene or reduced graphene oxide (rGO) nanosheets into viologens has been proved to be an effective approach to generate high performance EC composite materials with greatly enhanced cyclic stability and shortened EC response times [104,105,113]. Gadgil and co-workers have developed polyviologen (PV)-rGO nanocomposite films via a facile one-step electrocodeposition method where an aqueous dispersion of GO nanosheets is reduced during electropolymerization of a cyanopyridinium (CNP) monomer (Figure 14a) [104]. The as-prepared PV-rGO is highly stable due to the strong electrostatic and π–π stacking interactions between PV^2+^ and rGO as well as the well-established viologen cation–π electron interaction, which is a strong electrostatic interaction between positively charged cations with negatively charged electron cloud of π systems. The nanocomposite films deposited on the FTO substrates exhibit a high-contrast color change from transparent to purple with a low driving voltage of 0.6 V (Figure 14b). Compared to the PV/FTO film, the PV–rGO/FTO films demonstrate a remarkably higher switching stability. As shown in Figure 14c,d, the kinetic transmittance curves of PV–rGO/FTO films are consistent without any visible change over an entire scanning period of 8000 s under a switching step of 20 s. However, the transmittance of PV/FTO films at the colored state has decayed significantly (43%) compared to that of bleached state (3.5%), suggesting faster degradation of cation radical in pure PV films. The high durability of the color bearing viologen cation radical moiety in the composite films is asribed to the strong counterbalancing electrostatic interactions between PV and rGO, which is contrary to pristine PV film that does not have such interactions. The response time of the two films during kinetic switching experiments was also evaluated (Figure 14e). The bleaching and coloration time for the PV-rGO/FTO is 9 and 6 s, respectively, which is much faster than those for the PV/FTO films (17 and 12.5 s, respectively). Moreover, the calculated coloration efficiency value of the composite films (142 cm^2^ C^−1^) is also superior to that of the pristine PV/FTO films (98 cm^2^ C^−1^). The improved EC switching kinetics and coloration efficiency for the composite films can be attributed to the good carrier mobility and large surface area of rGO nanosheets, which facilitate the ion/charge transport within the bulk composite films.

**Figure 15:**
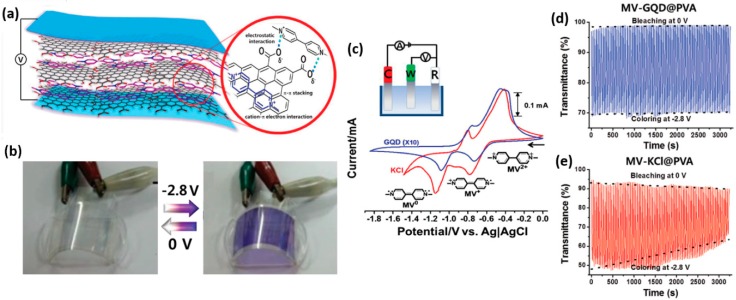
(**a**) Illustration of an electrolyte-free flexible electrochromic device of MV^2+^-GQDs. (**b**) Photographs for the reversible performance of a flexible MV^2+^-GQD EC device (containing 100 mM MV^2+^ with 8 mg mL^−1^ GQD in PVA) with ITO-on-PET. (**c**) Cyclic voltammetry of 5 mM MV^2+^ at an ITO electrode in an aqueous solution containing 8 mg mL^−1^ GQD (blue line) and 0.1 M KCl (red line) at a scan rate of 100 mV s ^−1^. Voltage-controlled transmittance changes by voltage switching between 0 (a bleached state) and −2.8 V (a colored state) for 50 mM MV^2+^ in an ECD at 550 nm. EC device of ITO-on-glass electrodes were prepared with: (**d**) 8 mg mL ^−1^ GQD in PVA and (**e**) 0.1 M KCl in PVA. Readapted with permission from WILEY-VCH [105].

Generally, the electrolytes used in an EC system are composed of ionic salts and liquid solvents. Metal cations are often combined with an organic solvent in the eletrolyte layer to serve as an ion conducting medium. The use of such eletrolytes sometimes poses the risk of decomposing the metal-ion-containing electrochromes at high voltages, leading to materials degradation and poor cycle stability [114]. Direct binding of electrochromes to electrolytes can give rise to a more stable EC platform, however, resulting in decreased switching properties and low coloration efficiency of the system [115]. To solve this problem, Hwang et al. recently developed a flexible electrolyte-free EC device utilizing electrostatically bonded graphene quantum dot (GQD)–viologen nanocomposites via physical mixing, in which the GQDs acted as a stable electrolyte-like charge transfer medium to facilitate the oxidation/reduction of methyl viologen (MV) [105]. As shown in Figure 15a, the composites are stabilized through strong intermolecular electrostatic interactions, π–π stacking and cation–π electron interactions. The two-electron transfer process of MV^2+^ was tested by cyclic voltammetry using an ITO electrode in the GQD solution in the absence of any electrolyte, and also in a commonly used electrolyte solution of KCl for comparison (Figure 15c). The position of redox peaks in the two systems perfectly matched, illustrating the typical EC behavior of MV^2+^ in the composites. A flexible EC device was fabricated based on the MV^2+^-GQD composite films without using any electrolyte (Figure 15b). The color can be reversibly changed from transparent to purple by applying pulsed voltages of 0 (bleached state) and −2.8 V (colored state) with a pulse width of 20 s. As shown in Figure 15d,e, compared to the conventional MV^2+^-KCl system, the electrolyte-free MV^2+^-GQD composites exhibit much more stable switching performance and good durabiltiy over a testing period more than 3200 s, which could be attributed to the strong non-covalent interactions between the MV^2+^ and GQDs.

**Figure 16:**
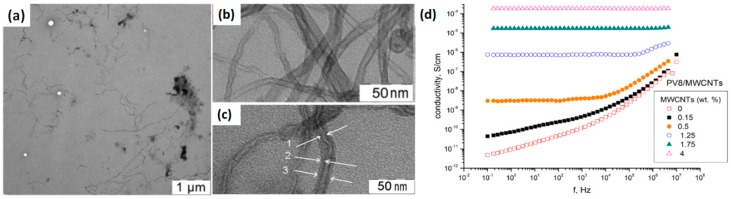
TEM images of (**a**) **PPDP**/MWCNTs dispersion in *N*,*N*-Dimethylmethanamide (DMF); (**b**) MWCNTs dispersion in DMF and (**c**) **PPDP**/MWCNTs dispersion in DMF obtained at high magnification. Arrows indicate: (1) MWCNTs (2) layer of **PPDP** coating the surface of MWCNTs (3) MWCNTs with the coated layer of **PPDP**. (**d**) Frequency dependencies of the real part electrical conductivity of the nanocomposites with different weight contents of MWCNTs at a **PPDP** concentration of 8 mg/mL. Readapted with permission from Elsevier B.V. [107,116].

Besides graphene nanosheets, carbon nanotubes can also be employed as conductive additives to improve the response times of EC polymers. The key challenge for processing of CNTs is their agglomeration problem caused by π–π stacking and Van der Waals attraction. Roman and coworkers recently developed composite EC materials from poly(4,4′-(1,4-phenylene)bis (2,6-diphenylpyridinium) triflate) (**PPDP**) and multiwalled carbon nanotubes (MWCNTs) via physical mixing [116]. The effective π–π stacking interaction between the benzene ring of **PPDP** with MWCNTs led to the stable adsorption of **PPDP** molecules on the surface of the MWCNTs, and the charged groups of **PPDP** also acted as functional units which enabled a stable dispersion. Figure 16a shows the TEM image of **PPDP**/MWCNTs composite material which demonstrates well separated carbon nanotubes and the absence of their aggregates. Under high magnification, it can be seen that the MWCNTs are uniformly coated with the **PPDP** polymer which is presented as a lighter layer with an average thickness of 6 nm on the nanotubes (Figure 16c, arrows indicated). Upon the addition of MWCNTs, the absorption bands and redox peak potentials of the polymer did not change. However, the resulting composite material exhibited significantly enhanced EC response times (200–400 ms). The coloration and bleaching times were decreased by 30 and 50%, respectively. The enhanced EC switching kinetics due to the improvement of electron transport in the bulk film is further demonstrated by the dielectric spectroscopy, displaying an increase in the electronic conductivity of **PPDP**/MWNTs composite films by 5 orders of magnitude in comparison with pure **PPDP** films (Figure 16d) [107].

#### 2.4.3. Dual EC Composites via Layer-By-Layer (LBL) Assembly

**Figure 17:**
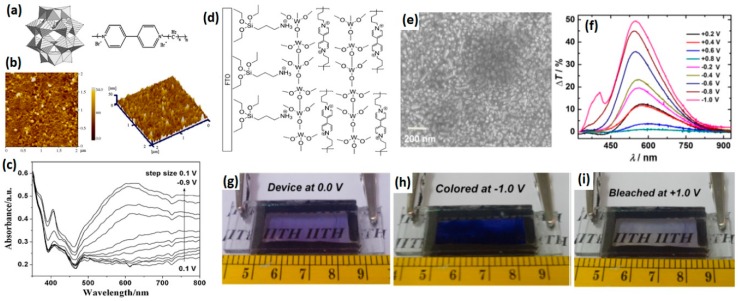
(**a**) The structure of tungsten phosphate (left) and PXV (right). (**b**) AFM images of a [P_2_W_18_/PXV]_5_ multilayer film on a silicon wafer. (**c**) Spectroelectrochemistry of [P_2_W_18_/PXV]_40_ composite film from +0.1 to −0.9 V with a step size of 0.1 V. (**d**) Structure of the WO_3_–PBV LbL film on FTO substrate. (**e**) SEM image of the surface of the deposited WO_3_–PBV LbL film. (**f**) Transmittance change versus wave length obtained under different potentials. Digital photos of the device based on the WO_3_–PBV composite film in (**g**) as fabricated, (**h**) in fully colored (E = −1.0 V), and (**i**) fully bleached (E = +1.0 V) states. Readapted with permission from Elsevier Ltd. [106,108].

Layer-by-layer (LBL) assembly is a facile processing technique that can combine two EC materials into a thin film with good control over thickness and composition, leading to a “dual electrochome” composite with multicolor switching and enhanced EC properties [117,118,119,120]. In addition to covalent attachment, inorganic EC materials can also be combined with viologens via LBL process. In 2009, Xu and co-workers reported a tunable multicolored composite film by LBL assembly of cationic poly(hexyl viologen) and anionic inorganic tungstophosphate (P_2_W_18_) nanoparticles (Figure 17a–c) [108]. Both P_2_W_18_ and poly(hexyl viologen) are cathodically coloring EC materials and cocurrently contributed to the coloration process. The composite films were found to switch from colorless to violet with a blue intermediate, due to the different coloration responses of two EC materials. In a most recent work, Sydam et al. reported a novel EC material based on poly(butyl viologen)/WO_3_ LBL films (Figure 17d–i) [106]. Due to highly efficient usage and short diffusion pathway of the ion/charge in the multilayer structure, the LBL nanocomposites films could realize quick color change (switching time ~1 s) under a potential of ±1.0 V with a high optical contrast of 49% and an outstanding coloration efficiency of 476 cm^2^ C^−1^.

## 3. Commercial Applications of Viologen-Based ECDs

The application of electrochromic technology has been extended to many fields, such as vehicles, displays, buildings, etc. Some products have been put into practical use, and the development of electrochromic devices has presented significant commercial value. The first EC display based on viologen was invented by the Philips Laboratories [121,122] in the early 1970s, which employed heptyl viologen (HV) as the EC species, leading to a final ECD with a high contrast ratio of 20:1, a rapid erasing time (10–50 s) and a cyclic durability of more than 105 cycles. Based on the same viologen, the IBM laboratories also developed an ECD display which was a 64 × 64 pixel integrated device with eight levels of grey tone on a one-inch-square silicon chip [123]. At the same time, a data display device was marketed by Imperial Chemical Industries Limited (ICI) using the arylsubstituted viologen ‘cyanophenyl paraquat’ with higher color efficiency and faster switching time [124]. Although the development of these devices was not continued due to the competition of liquid-crystal display (LCD) technique, they still demonstrated a great potential in the production of large-size devices.

**Figure 18:**
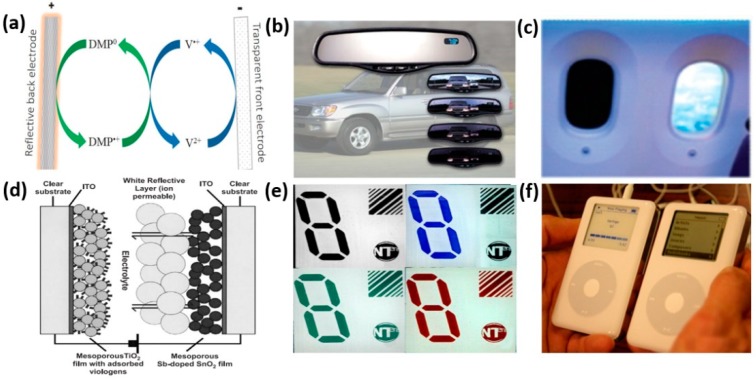
(**a**) Schematic representation of the redox cycles occurring within the Gentex automatic dimming rear view mirror. (**b**) A photograph of Gentex auto dimming mirror device. (**c**) A photograph of electrochromic windows on the Boeing 787 Dreamliner. (**d**) Cross-section structure of a NanoChromics display device. (**e**) A photograph of colored NanoChromics devices. (**f**) A photograph of ipod prototypes with NanoChromics display (left) and LCD display (right). Reproduced with permission from Elsevier B.V. [127].

The Gentex automatic dimming rear view mirror (Figure 18b) is the most widespread commercial ECD in the market [125,126]. With this sophisticated night driving safety accessory, special sensors and electronic circuitry can detect glare and automatically dim the mirror accordingly, which can eliminate dangerous glare from headlights of rear-approaching vehicles. This system functions based on the solution electrochromism using a type II EC material. As shown in Figure 18a, the mirror is composed of an ITO coated-glass electrode and a reflective metallic electrode, with a solution containing cathodically colored viologen and anodically colored 9,10-dimethylphenazine (DMP). After switching on the mirror, the two species migrate to their respective electrodes. The viologen is electro-reduced at the cathode to form the colored viologen^•+^ radical cation, while the neutral DMP is electro-oxidized at the anode and provides a complementary color to the viologen. Once the dual electrochromic coloration process starts, the products diffuse away from their corresponding electrodes and meet in the intervening solution, where their mutual reaction occurs to regenerate the original uncolored species. After extensive application of the automatic dimming rear view mirror in over 220 models of cars worldwide, the Gentex Corporation further worked with PPG Aviation Industries in 2005 to install electrochromic windows on the Boeing 787 Dreamliner (Figure 18c). Passengers can adjust the brightness of the portholes with a five-speed button to make the cabin more comfortable. In July 2008, the electrochromic portholes were successfully brought into operation.

Another well-known viologen-based ECD is the ‘paper quality’ NanoChromics^TM^ display developed by Ntera Ltd. In 2003, which can achieve 4 times higher contrast than ordinary black and white LCD panels [127,128]. Moreover, it does not need a backlight and will not consume power as long as the display content does not change, leading to 10 times more battery driving time than ordinary LCD panels. As shown in Figure 18d, the NanoChromic device comprises two high-surface-area mesoporous metal oxide (TiO_2_ and SnO_2_:Sb) electrodes modified with phosphonated viologens and phenothiazine, respectively. The adsorption of the viologens enhances switching speed as the switching process is not limited by diffusion of viologens. The counter electrode of mesoporous Sb:SnO_2_ acts as a mediator of the redox reaction and efficiently stores the charge. Through modification of viologen with various substituents, a broader color range can be achieved, as shown in Figure 18e. The trial experiments of this device showed a color efficiency of 170 cm^2^ C^−1^ and stability over 10,000 switch cycles. The display was said to be ultra-fast, owing to its faster switching speed, at which an absorbance change of 0.6 was attained in just one second. The application of the NanoChromics™ display was demonstrated in a converted iPod (Figure 18f) and ‘consumer product reference designs’ for digital clocks and an eight-digit calculator. The prototype display was also stated to be applicable to several products including windows, mirrors, flexible electronic displays, dimmable window laminates, games and toys.

## 4. Conclusions and Outlook

In conclusion, this review gives an overview of chemical structures, preparation and EC properties of various viologen-based materials, including small molecular and polymeric viologens, viologen-functionalized conjugated polymers and viologen-based organic/inorganic composite materials. Their ECD performance including colors, transmittance change during coloration and bleaching process, bleaching and coloration time and coloration efficiency, etc., was also summarized. Apart from the variations on the nitrogen substituents, additional bridging or introduction of heteroatoms to the viologen molecule can further enhance their electronic and photophysical properties. The selection of suitable complementary redox species and conducting polymer backbones to integrate with viologens is essential to enhance the performance of the ECDs or offer additional colors in addition to the characteristic blue/violet of viologens. To improve cyclic reversibility and achieve fast response times for practical applications, various viologen-based organic/inorganic composite materials have been developed through chemical anchoring or physical interactions. To overcome the shortages of liquid electrolyte, gel/ionic liquid-based electrolytes were successfully employed, and binding electrochromes to electrolytes or even constructing electrolyte-free ECDs could lead to more stable EC platforms. Significant progress has been made on viologen-based EC materials and their ECDs in the past two decades, but challenges for viologen-based materials, such as long-term stability and switch speed, are not fully addressed. Future developments of viologen-based EC technology may be focused on finding more sophisticated viologen-based molecules, polymers or composites with more color availability, longer stability, faster switch speed and higher coloration efficiency, so as to meet various industry applications.

## Figures and Tables

**Table 1:** ECD performance of selected small viologen molecules and their derivatives.

EC Material	Color (O) ^a^	Color (R) ^b^	λ_max_^c^ (nm)	V_b_/V_c_^d^ (V)	∆T ^e^ (%)	t_b_/t_c_^f^ (s)	Stability (cycles)	CE ^g^ (cm^2^ C^−1^)	Ref.
**V5**	colorless	Green or red	600/500	0/−1.4 or −1.8	30	-	6000	-	[20]
**V6/V4**	colorless	black	615	0/0.5	65	3.5/2.8	10,000	-	[21]
**V7**	colorless	Dark violet-blue	605	1.5/−1.5	52	2.1/2.2	2000	533	[22]
**V8**	Slight yellow	Deep blue	607	0/0.9	83	-	-	315	[23]
**V9**	Greenish yellow	amaranth	520	0/−1.0	51	2.1/4.5	10,000	301	[24]
**MV1**	colorless	magenta	520	0/−1.5	68	1/1.5	1000	875	[25]
**MV3** (Ar=PV)	colorless	pink	512	0/−1.5	76.9	9.5/56.7	1000	-	[26]
**MV3** (Ar=NV)	colorless	purple red	544	0/−2.5	74.1	18.4/41.4	1000	-	[26]
**MV3** (Ar=BV)	colorless	deep violet-blue	592	0/−2.4	69	24/38.6	1000	-	[26]
**MV4**	bright red	blue/violet	635	0.5/−0.8	33	1.9/1.8	-	-	[27]
**MV6** (R=vinyl)	colorless	Red brown	522	1.6/−1.6	75	0.79/0.48	1000	645	[28]

NOTE: ^a^ Color at oxidized state; ^b^ color at reduced state; ^c^ maximum absorbance wavelength; ^d^ bleaching (V_b_) and coloration potential (V_c_); ^e^ transmittance change during coloration and bleaching process; ^f^ bleaching (t_b_) and coloration time (t_c_); ^g^ coloration efficiency.

**Table 2:** ECD performance of selected polyviologens.

EC Material	Color (O) ^a^	Color (R) ^b^	λ_max_^c^ (nm)	V_b_/V_c_^d^ (V)	∆T ^e^ (%)	t_b_/t_c_^f^ (s)	Stability (cycles)	CE ^g^ (cm^2^ C^−1^)	Ref.
**PV I**	Colorless	blue	700	0/−1.0	34	0.26/0.39	1000	204	[64]
**PV II**	Colorless	blue	650	0/−1.0	28	0.54/2.18	1000	117	[64]
**PV I**/PSS	Colorless	blue	700	0/−1.0	34.7	0.31/0.26	50	-	[62]
**PV I**/PVCa	Slight yellow	blue	700	−0.5/−1.2	40	13.5/1.2	50	-	[61]
**V3**/PEDOT:PSS	Colorless	blue	525	0.5/−0.9	82.1	2.2/3.1	-	-	[48]
PBV ^h^/PB ^i^	colorless	purplish-blue	650	−1.0/0.7	65	9.4/2.0	4000	163	[49]
**CV1**	colorless	Dark blue	625	0/−1.0	-	-	-	97	[69]
**CV4**	colorless	Dark violet	610	−0.6/1.0	39	7/5	1000	305	[70]
**CV8**	colorless	blue	610	1.0/−1.0	65	3.7/8.4	-	-	[71]
**CV10** (R=PM)	Pale yellow	Dark cyan	740	0/1.05	64	-	-	-	[72]
**CV10** (R=BP)	Pale yellow	Dark cyan	742	0/1.05	50	-	-	-	[72]

NOTE: ^a^ Color at oxidized state; ^b^ color at reduced state; ^c^ maximum absorbance wavelength; ^d^ bleaching (V_b_) and coloration potential (V_c_); ^e^ transmittance change during coloration and bleaching process; ^f^ bleaching (t_b_) and coloration time (t_c_); ^g^ coloration efficiency; ^h^ poly(butylviologen); ^i^ prussian blue.

**Table 3:** ECD performance of various selected viologens-based composite materials.

EC Material	Color (O) ^a^	Color (R) ^b^	λ_max_^c^ (nm)	V_b_/V_c_^d^ (V)	∆T ^e^ (%)	t_b_/t_c_^f^ (s)	Stability (cycles)	CE ^g^ (cm^2^ C^−1^)	Ref.
BPV ^h^/meso-nc-TiO_2_^i^	colorless	deep blue	608	2.5/−2.5	53	0.9/2.1	100	-	[88]
BPV/nc-TiO_2_^j^	colorless	deep blue	608	0/−1.5	55	1/1	10,000	170	[89]
PBT ^k^/nc-TiO_2_	brown yellowish	dark blue	600	2.0/−2.0	64.8	0.72/0.6	8000	912	[94]
PBT/nc-TiO_2_	yellow green	deep black	570	0.5/−1.5	60	4.8/3.0	120,000	-	[97]
**V1**/ZnO NTs ^l^	colorless	blue	608	3.0/−3.0	70	3/6	500	94	[100]
**V1**/ZnO NWs ^m^	colorless	blue	608	2.0/−2.0	46	0.14/0.17	200	196	[98]
DDV ^o^/ITO NPs ^n^	white	violet	512	0/−0.6	15	0.38/0.5	500	140	[101]
DDV/SnO_2_ NPs	white	purple	510	0/−0.5	32	0.53/1.5	150	-	[103]
**PV I**/rGO ^p^	colorless	purple	525	0/−0.6	52	9/6	400	142	[104]
**V1**/GQD ^q^	colorless	purple	550	0/−2.8	29	39/26	3000	65	[105]
WO_3_/PBV/RP ^r^	colorless	deep blue	580	1.0/−1.0	49	0.7/0.8	500	476	[106]
**PPDP**/MWCNTs	colorless	deep blue	700	0.3/−1.0	52	0.35/0.32	-	-	[107]
**V3**/P_2_W_18_	colorless	violet	600	0.1/−0.9	42.7	2.0/5.8	200	-	[108]
PXV ^s^/SQ NPs ^t^	orange	dark purple-blue	550	−0.7/−1.3	50	3.5/3	-	205	[109]

NOTE: ^a^ Color at oxidized state; ^b^ color at reduced state; ^c^ maximum absorbance wavelength; ^d^ bleaching (V_b_) and coloration potential (V_c_); ^e^ transmittance change during coloration and bleaching process; ^f^ bleaching (t_b_) and coloration time (t_c_); ^g^ coloration efficiency; ^h^ bis-(2-phosphonoethyl)-4,4′-bipyridinium dichloride; ^i^ mesoporous nanocrystalline TiO_2_; ^j^ nanocrystalline TiO_2_; ^k^ 1,4-bis[((*N*-phosphono-2-ethyl)-4,4′-bipyridinium)-methyl]-benzene tetrachloride; ^l^ nanotubes; ^m^ nanowires; ^n^ nanoparticles; ^o^ 1,1′-didodecyl-4,4′-bipyridinium; ^p^ reduced graphene oxide; ^q^ graphene quantum dots; ^r^ ruthenium purple; ^s^ 1,1′-bis[3-(trimethoxysilyl)propyl]-4-4′-bipyridinium iodide; ^t^ silsesquioxane nanoparticles.
